# The Co-Occurrence of Autism Spectrum Disorder and Aarskog–Scott Syndrome in an Accomplished Young Man

**DOI:** 10.3390/pediatric17040073

**Published:** 2025-07-08

**Authors:** Raisa S. Romanova, Oksana I. Talantseva, Katerina V. Lind, Victoria A. Manasevich, Julia E. Kuznetsova, Elena L. Grigorenko

**Affiliations:** 1 Center for Cognitive Sciences, Sirius University of Science and Technology, 354340 Sirius, Russia; rayaromanova@me.com (R.S.R.); katerina.lind@gmail.com (K.V.L.);; 2 Association of Psychiatrists and Psychologists for Evidence-Based Practice, 117628 Moscow, Russia; talantcevaoksana@gmail.com; 3 Department of Psychology, University of Houston, Houston, TX 77204, USA; 4 Department of Molecular and Human Genetics, Baylor College of Medicine, Houston, TX 77030, USA; 5 Child Study Center, Yale University, New Haven, CT 06519, USA

**Keywords:** Aarskog–Scott syndrome, AAS, faciogenital dysplasia, autism spectrum disorder, ASD, attention deficit/hyperactivity disorder, intellectual disability

## Abstract

**Objectives/Background:** Aarskog–Scott syndrome (AAS), also known as faciogenital dysplasia, is a rare X-linked genetic disorder primarily characterized by its diverse physical manifestations. Previous evidence suggests a potential association between AAS and neurodevelopmental disorders, including autism spectrum disorder (ASD). **Methods:** This case study presents a male adolescent with ASD and a novel genetic variant in *FGD1* underlying AAS. We conducted comprehensive clinical, genetic, and behavioral assessments to characterize the neurodevelopmental presentation. Moreover, we examined the existing literature on AAS and comorbid neurodevelopmental disorders. **Results:** The patient demonstrated features consistent with both AAS and ASD, presenting with characteristic physical features of AAS and meeting diagnostic criteria for ASD on both ADI-R and ADOS-2. Cognitive assessment revealed above-average nonverbal IQ (Leiter-3, NVIQ = 115), while adaptive functioning was notably impaired (Vineland composite score = 65). Executive function deficits were identified through several assessments, though ADHD diagnostic criteria were not met. The literature review considered 64 studies, including 151 individuals with AAS. ASD was observed in 4.0%, Attention Deficit/Hyperactivity Disorder (ADHD) in 10.6%, and Intellectual Disability (ID) in 14.2% of cases. **Conclusions:** The combination of ASD with preserved nonverbal intelligence but impaired adaptive functioning in this AAS case demonstrates the complex neurodevelopmental manifestations possible in this rare genetic condition. The prevalence of neurodevelopmental disorders among people with AAS may be higher than their prevalence in the general population. However, a comprehensive assessment of developmental progress was rarely performed in previous studies, which may lead to systematic underestimation of co-occurring neurodevelopmental difficulties in AAS.

## 1. Introduction

Aarskog–Scott Syndrome (AAS; OMIM #305400), also known as faciogenital dysplasia, is a rare genetic disorder with an estimated prevalence of 1 in 25,000 live births [1]. AAS is characterized by a heterogeneous clinical presentation, varying in its severity and combination of symptoms among affected patients [2]. In 1993, these features were segmented into a set of symptoms by Teebi [3]. The most common phenotypic manifestations of AAS include a short stature, craniofacial dysmorphism (i.e., hypertelorism, ptosis, anteverted nares, and long philtrum), and skeletal (i.e., brachydactyly, clinodactyly, short/broad hands, interdigital webbing) and genital anomalies (i.e., shawl scrotum). The presence of three or more classical signs could suggest a clinical suspicion of AAS. Importantly, AAS may also be accompanied by several secondary, additional, and other features [4]. Although AAS primarily affects males due to its X-linked recessive inheritance pattern, females may also be carriers of the syndrome but are typically asymptomatic or have milder symptoms [4].

To date, only one causal gene, *FGD1* (OMIM #300546), has been identified for AAS. Being a member of the guanine nucleotide exchange factor, *FGD1* has been reported to contribute to embryonic development in mammals, participating directly in multiple signaling pathways, including gene expression, cytoskeleton organization, cell polarization, and cycle progression ([2,4,5] for a review). Hence, mutations in the *FGD1* gene may lead to developmental disorders, affecting specific skeletal structures, including elements of the face, cervical vertebrae, and distal extremities [6]. Overall, at least 61 altered variants of *FGD1* associated with AAS have been characterized [1]. Although a correlation between these variants and the spectrum of clinical expression in AAS has been hypothesized and intensively investigated, no clear phenotype–genotype correlation has been revealed [7]. Notably, the majority of AAS cases were reported before the advent of molecular testing. Therefore, the reported mutations describe only about 20% of the registered cases [7,8]. Given the overlapping symptoms of AAS with a number of other conditions, i.e., the Noonan, SHORT, and Robinow syndromes, as well as pseudohypoparathyroidism, genetic testing is a critical element of comprehensive AAS diagnostics [1].

While AAS is primarily recognized for its physical manifestations, additional and other categories of symptoms, of developmental delay and ADHD, are also listed [4]. In addition, there are a few reports of AAS being accompanied by other neurodevelopmental, behavioral, and mental disorders, including autism spectrum disorder, ASD [9,10]; intellectual disability, ID [10]; oppositional defiant disorder, ODD [11]; and learning disabilities, LD [12]. Intelligence ranges from normal to severe ID. Logie and Porteous [13], in a sample of 21 boys, observed a normal IQ distribution consistent with the general population. The IQ ranged from 69 to 128, with an average score of 106. However, other studies suggest an increased frequency of ID among individuals with AAS, up to 30% [13].

The potential link between AAS and neurodevelopmental disorders is still being investigated. It has been suggested that rare genetic variants in the *FGD1* gene could lead to cortical malformations, including polymicrogyria and focal cortical dysplasia [14], or to a disruption in normal signaling pathways [15]. The abnormal cortical functioning may interfere with normal brain development and, therefore, contribute to cognitive and behavioral phenotypes associated with AAS.

However, given the sporadic and inconsistent findings on neurodevelopmental alterations associated with AAS and their frequency, the current study aimed to contribute to these reports by providing a description of a case study with a comprehensive clinical and cognitive assessment of the literature. To accomplish this aim, we present a case of a 17-year-old male patient with AAS with an evaluation of accompanying neurodevelopmental disorders. We also summarize the existing literature on neurodevelopmental disorders that have been reported in AAS.

## 2. Materials and Methods

### 2.1. Genetic Testing

Whole genome sequencing (WGS) was performed in an external certified clinical laboratory (Evogen Lab, Moscow, Russia) using a PCR-free paired-end sequencing approach with ≥30x average genome coverage. The bioinformatic analysis included variant filtering and pathogenicity scoring per ACMG guidelines [16] and was interpreted by a board-certified clinical geneticist. The raw data were not available to our research team; interpretation was based on medical documentation provided by the patient’s parents.

Segregation analysis was not available; however, the clinical laboratory recommended testing the mother to assess whether the identified variant was inherited or de novo. Genetic analysis was performed on DNA extracted from venous blood.

### 2.2. Clinical and Behavioral Assessment

Clinical and behavioral assessments were conducted at the Center for Cognitive Sciences, Sirius University of Science and Technology (Sirius, Sochi, Russia). To evaluate the key manifestations of ASD in the present case, we employed the Autism Diagnostic Interview–Revised (ADI-R) [17] and the Autism Diagnostic Observation Schedule, 2nd Edition, Module 3 (ADOS-2) [18]. Both methods were administered by trained clinical psychologists. For a comprehensive assessment of intelligence, we used the Universal Nonverbal Intelligence Test, Second Edition (UNIT-2) [19], and the Leiter International Performance Scale, 3rd Edition (Leiter-3) [20]. To assess adaptive functioning, the Vineland Adaptive Behavior Scales, 2nd Edition (Vineland-II), were utilized [21]. Because conditions such as affective disorders and ADHD are common in ASD [22] and there is evidence of an association between AAS and both executive function deficits and ADHD [11], the following instruments were also used: the Child Behavior Checklist for ages 6 to 18 (CBCL/6-18) [23]; The Behavior Inventory of Executive Function, 2nd Edition (BRIEF2) [24]; and the Conners 3rd Edition [25]. Diagnostic decisions were made according to DSM-5-TR criteria [26].

## 3. Results

### 3.1. Case Presentation

A male patient (ID UN319) was born after 40 weeks of gestation to non-consanguineous parents, with the mother (29 years old) of Russian descent and the father (42 years old) of Armenian descent. The patient is the eldest of two sons, with a younger sibling who was not assessed clinically but is reported by the family to be developing typically. The pregnancy was unplanned and complicated by severe edema, hypertension, and elevated maternal stress levels. No infections, medication use, smoking, or intake of alcohol or drugs during pregnancy were reported. The final weeks of gestation were complicated by preeclampsia. Prenatal ultrasound revealed fetal chondrodystrophy. Labor, which occurred at term, was prolonged (16 h) due to insufficient cervical dilation. At birth, the patient’s weight was 3890 g (86th percentile), height was 52 cm (87th percentile), and Apgar scores were 8 and 9 at 1 and 5 min, respectively. Immediately after birth, the patient exhibited increased muscle tone.

Further development was characterized by the features associated with the ASD phenotype. The patient exhibited advanced motor development, standing at 5 months and walking at 8 months, and displayed a specific gait pattern and tiptoe walking. He also showed a lack of response to his name and engaged in stereotyped, repetitive play. During his kindergarten years, the patient experienced language and speech difficulties. The mother reported sleep disturbances, with the patient sleeping only 4–5 h a day and swallowing issues that necessitated food chopping. No difficulties were recorded at elementary school. However, in secondary school, the patient had a brief period when he attended classes with a tutor to support him in the classroom. There were no reported conflicts with teachers or peers. Upon entering a technical college at the age of 16, the patient developed academic performance issues that had not manifested previously. College instructors also noted behavioral peculiarities that hindered the patient’s learning progress. Psychological assessments showed that the patient exhibited social “clumsiness” in various public situations, struggling to understand his role in certain types of social relationships and communication contexts. He also demonstrated a low level of adaptive functioning and experienced difficulties with executive functions such as planning, regulation, and control, which significantly complicated the educational process.

The patient was referred to our team at the age of 17 years and 1 month because of his communication problems, associated academic difficulties, and concerns about a genetic disorder. Physical examination revealed that the patient’s facial features were consistent with AAS, including an antimongoloid slant of the eyes, telecanthus, epicanthus, widow’s peak hairline, attached earlobes, a broad neck with pterygium, low posterior hairline, and hyperextension of the distal phalanges of the fingers. His sexual development was age-appropriate, with a shawl scrotum observed during the physical examination.

### 3.2. Molecular Findings

Whole genome sequencing identified a two-nucleotide insertion in the non-coding region of exon 11 of the *FGD1* gene (NC_000023.11:n. 54481961_54481962insAT) in a hemizygous state. Although this variant has not been previously reported in genome browsers, several hemizygous variants in *FGD1* have been implicated in AAS and syndromic X-linked intellectual disability type 16 [2,15,27]. Computational predictive programs (SpliceAI, SPIP, Squirl) indicated that this sequence variant disrupts the splice acceptor site. According to the ACMG guidelines, the criteria applied to this variant include PVS1, PM2, PP3, and PP4. The identified nucleotide sequence variant was considered pathogenic and causative for AAS based on cumulative evidence. Following WGS, the presence of this genetic variant was confirmed with Sanger sequencing.

### 3.3. Clinical and Behavioral Assessment

#### 3.3.1. ASD Diagnostics

The ADI-R results were above the clinical cut-off values for ASD in all diagnostic domains (Table A1). Throughout the ADOS-2 assessment, the patient demonstrated the ability to maintain a structured reciprocal conversation, but with a slightly limited set of gestures and poorly modulated eye contact. Appeals were limited to personal needs or areas of increased interest. He responded to most social situations, but his reactions were somewhat limited and socially awkward. Some words and phrases tended to be repetitive and formal. The patient occasionally turned to specific topics and patterns of interest, and repetitive forms of behavior were observed. Unusual sensory interests were not revealed. Hands, fingers, or other movements specific to ASD were absent. Hence, the total overall ADOS-2 score corresponded to an autistic profile with moderate severity of symptoms (Table A2).

#### 3.3.2. Cognitive Development

The patient’s nonverbal performance, according to Leiter-3, was above average (NVIQ = 115). Within the profile, his cognitive abilities varied from average results in the Figure Ground and Form Completion subtests to very high results in the Sequential Order subtest. His memory functions were within the average range, while the results of subtests evaluating his attention abilities were slightly below average, indicating the presence of mild difficulties (Table A3). The UNIT-2 assessment yielded similar results. The patient’s overall IQ was above average (NVIQ = 113). His cognitive abilities also varied from average scores in the Symbolic Memory, Nonsymbolic Quantitative, Analogies, and Number Series subtests to a superior level in the Cube Design subtest (Table A4).

#### 3.3.3. Adaptive Functioning

The patient’s Adaptive Behavior Composite score of 65 (according to the mother’s report) classifies his general adaptive functioning as low. Within the profile domains, his standard scores varied from low to average results. The lowest scores occurred in the Daily Living Skills and Socialization domains. A significantly profound decrease was also identified in his writing and reading skills (scored as low). Subtests assessing maladaptive behavior revealed an increased risk of externalizing and internalizing disorders (Table A5).

### 3.4. Assessment of Comorbid Disorders

The CBCL/6-18 form was completed by the patient’s mother. In terms of the Competence scale, his total score was in the normal range. However, the School subscale score was in the subclinical range, mostly because of difficulties in relationships with teachers and classmates. The overall result for the Syndrome scale was within the clinical range, with the most profound increase in Social Problems following the Thought Problems subscale. Along with this, his score for the combined Internalizing Behavior scale was also increased. Thus, according to these DSM-5-oriented scales, a risk of anxiety problems and ADHD was revealed (Table A6).

The BRIEF2 assessment was completed by both parents and by the patient himself. Despite the quantitative differences between the reports, all of them indicated a pronounced impairment in the patient’s executive functions. Contrary to his parents, the patient had not identified any problems associated with the Self-Monitor scale (Table A7, Table A8 and Table A9).

The Conners-3 assessment, according to the mother’s report, showed elevated scores for the Learning Problems and Peer Relations scales. At the same time, the ADHD, Conduct Disorder, and Oppositional Defiant Disorder diagnostic scales had not reached their at-risk cut-offs (Table A10).

## 4. Discussion

The current case study contributes to the growing body of literature on the cognitive, behavioral, and mental health manifestations associated with AAS. We present an in-depth assessment of a 17-year-old male with AAS who was diagnosed with ASD and demonstrated impairments in executive function. His nonverbal intelligence was determined to be in the above-average range, with some abilities corresponding to the superior level. However, his adaptive functioning was assessed as low due to his clinically impaired performance during daily living and socialization, presumably because of ASD.

To contextualize this case and use examples of such contextualization that are available in the literature [28,29], we utilized the PRISMA protocol for scoping reviews (see Appendix B) as our framework [30].

A total of 151 male patients were identified in the literature and were eligible to be included in the synthesis. These patients were from 25 countries sampled from Europe (N = 57), North America (N = 52), Asia (N = 38), and South America (N = 4). Excluding three studies (Assumpcao et al., 1999; Taub et al., 2008; Melnick et al., 1976) [9,31,32], the race and ethnicity characteristics of the patients were not reported in most studies. Coding based on the provided information about nationality, country of origin, and photos yielded the following distribution: Asian (N = 26), Black and African American (N = 1), Hispanic and Latino (N = 14), White (N = 72), Middle Eastern and North African (N = 10); in 28 cases, coding was not possible. The ages of the participants varied from 1.5 to 60 years (M = 12.8, SD = 11.0).

ASD was reported in six cases (4.0% of the total sample). In an additional eight cases (5.3%), autistic features were described, but no information was provided about the corresponding clinical assessments or subsequent diagnoses. None of the studies employed gold-standard, autism-specific diagnostic tools (e.g., ADOS, ADI), and among the ASD-diagnosed cases, the diagnostic criteria were explicitly reported in only one article [9]. Although comprehensive ASD assessment was uncommon among the reviewed studies, the available data suggest that the possible prevalence of ASD in AAS patients could be as high as 15.3%. While prevalence estimates of ASD in the general population are highly variable—with the most recent CDC report indicating rates as high as 3.1%—recent meta-analyses place the global prevalence between 1.0% and 1.2% [33,34], which is substantially lower than the rates found in the AAS population. At the same time, it is important to acknowledge that, given the methodological limitations of the existing literature and the lack of specific focus on ASD assessment in individuals with AAS, these findings may be biased toward overdiagnosis. Further rigorous investigation is needed to clarify the true prevalence of ASD in this population.

Intellectual development was assessed as in the normal range for 76.3% of cases (100/131). Among them, 12 individuals were within the low average, 17 in the average, 5 in the high average, and 4 in the superior category. However, for most of them, it was not possible to specify the intelligence category. Thus, 9.2% of individuals (12/131) had borderline intellectual functioning. ID was identified in 14.5% of cases (20/131). Among them, 13 had mild and 4 had severe ID (for 3 cases, the category was not identified). IQs were reported for 52 cases. They varied from 59 to 128, with an average quotient equal to 89.1 (SD = 18.7, Mod = 84.0). Excluding 43 cases, the diagnostic instruments employed to assess intelligence were not reported.

ADHD was also reported in the studied AAS patients. It was diagnosed in 10.6% of cases, and the additional 2.0% (described in [35,36,37]) were classified as at-risk due to specific ADHD-associated features, like deficits in attention and executive function, as well as hyperactivity. Compared to the meta-analysis [4], in which only cases with genetically confirmed AAS were included, the prevalence of ADHD in AAS patients was estimated at 10.3%, close to our results and higher than in general. Thus, according to systematic reviews and meta-analyses, ADHD is prevalent among 7.6% of children and 2.6% of adults [38,39]. As such, ADHD is listed under the “Other” category of clinical features possibly associated with AAS. Interestingly, according to this meta-analysis, the prevalence of ADHD in AAS patients is higher when estimates for a number of other phenotypic features related to additional, secondary, and even primary criteria of ADHD are considered.

The available studies suggest that the prevalence of ASD and ADHD in individuals with AAS may be higher compared to the general population. However, these neurodevelopmental disorders are often not the primary focus of AAS case reports, and standardized diagnostic assessments are infrequently used. This lack of comprehensive evaluation may lead to an underestimation of the true prevalence of these comorbidities in AAS.

It is important to know, however, that the studies considered in the current literature review have a number of weaknesses. First of all, there are very few AAS studies in which the comprehensive evaluation of neurodevelopmental disorders was performed. While the assessment of intellectual development is most common, the evaluation of social, communicative, language, and executive function development seems to be exclusive and mostly presented by reports that focus specifically on deficits in one domain. Moreover, many of the existing reports lack details on the specifics of the evaluated constructs and the corresponding assessments. All of this reduces the quality of the extracted data used for our scoping review. It may also imply that the observed frequencies are underestimated and that the increased quality of studies and added focus on neurodevelopmental disorders could result in higher estimates.

Our findings are consistent with previous case reports describing AAS patients with ASD and normative IQ [9,40]. While intellectual development in AAS has been more commonly studied, with IQ estimates ranging from severe intellectual disability to superior intelligence [13,41], there has been a limited focus on other neurodevelopmental comorbidities such as ASD and ADHD.

In the presented case, despite noted executive function deficits, which are common in both ASD and ADHD [42], a diagnosis of ADHD was not confirmed based on the collected data. We hypothesize that the observed executive dysfunction is more likely associated with ASD than a distinct manifestation of ADHD.

These findings highlight the importance of routine comprehensive neurodevelopmental assessments in individuals with AAS. Early detection of and intervention in neurodevelopmental disorders are crucial for optimizing developmental outcomes, as a lack of timely diagnosis and management can lead to significant delays in achieving cognitive and academic milestones, increased risk of comorbid mental health difficulties, reduced adaptive functioning and independence, and negative impacts on social integration and peer relationships [43,44]. In cases of AAS accompanied by developmental disabilities, appropriate special education accommodations and targeted interventions may be necessary.

The research presented here has a number of limitations. First, the scoped articles were not assessed for study quality. Therefore, all conclusions based on them should be interpreted with caution. Second, given the lack of standardized diagnostic instruments in Russia relevant for individuals in our patient’s age range, we did not assess his verbal IQ or language development. Considering the published studies on the intellectual profiles of ASD patients (NVIQ > VIQ), we presume that for this patient, the total IQ could significantly differ from his NVIQ. Thus, our reference to the patient’s intelligence as above average may be inaccurate. Additionally, brain imaging was not performed as part of the clinical evaluation. This may limit our ability to correlate neurocognitive findings with potential structural abnormalities.

Nevertheless, this case study and the contextualizing literature review contribute to clinical and translational research on AAS by highlighting the importance of comprehensive neurodevelopmental assessment of individuals with this syndrome, especially longitudinally. Moreover, the case study contributes to the data on the behavioral manifestations of the rare genetic condition, AAS. Finally, it reports a previously unknown genetic variant associated with AAS. In summary, given the heterogeneity of the etiology and manifestation of AAS, the literature on it is likely to grow as more cases become available that are, hopefully, better characterized, both molecularly and behaviorally.

## Figures and Tables

**Figure A1:**
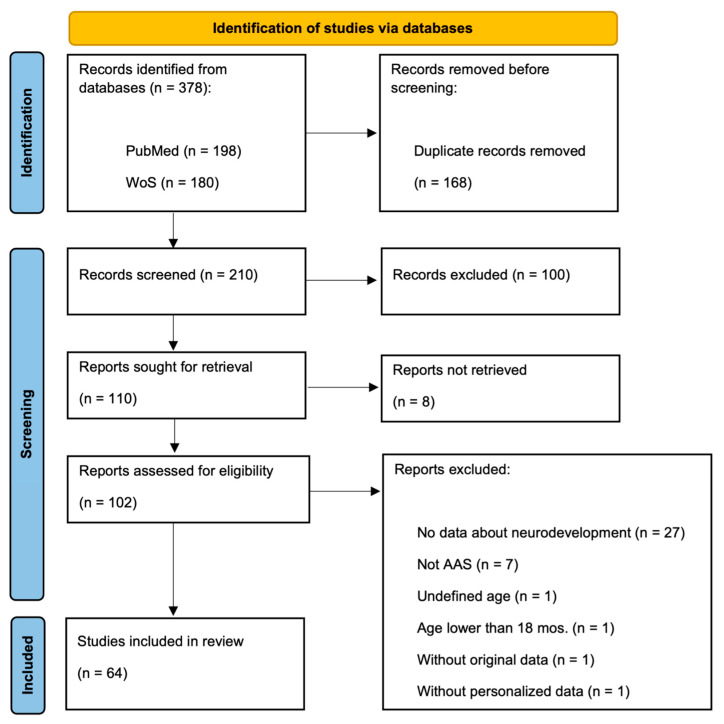
Flow diagram of the search strategy.

## Data Availability

The authors confirm that the data supporting the findings of this study are available within the article.

## Supplementary Materials

The following supporting information can be downloaded at: https://www.mdpi.com/article/10.3390/pediatric17040073/s1, Table S1: Summary of characteristics of reported patients with Aarskog–Scott syndrome. References [2,3,5,9,10,11,12,13,14,15,27,31,32,35,36,37,40,45,46,47,48,49,50,51,52,53,54,55,56,57,58,59,60,61,62,63,64,65,66,67,68,69,70,71,72,73,74,75,76,77,78,79,80,81,82,83,84,85,86,87,88] indicate studies included in the review (see Supplementary Table S1).

## Appendix A

**Table A1:** Results of assessment of core symptoms of autism using ADI-R.

ADI-R Domain	Score	Threshold
Qualitative impairments in reciprocal social interaction	25	10
Qualitative impairments in communication (verbal)	9	8
Repetitive behaviors and stereotyped patterns	10	3

**Table A2:** Results of assessment of core symptoms of autism using ADOS-2.

ADOS-2 Scale	Item	Score
Social affect (SA)		
*Language and communication*		
Reporting of events	A-7	0
Conversation	A-8	0
Descriptive, conventional, or informational gestures	A-9	1
*Reciprocal social interaction*		
Unusual eye contact	B-1	2
Facial expressions directed to others	B-2	1
Shared enjoyment in interaction	B-4	1
Quality of social overtures	B-7	1
Quality of social response	B-9	1
Amount of reciprocal social communication	B-10	0
Overall quality of rapport	B-11	0
***SA total:***	***7***	
Restricted and repetitive behavior (RRB)		
*Play, stereotyped behaviors, and restricted interests*		
Stereotyped/idiosyncratic use of words or phrases	A-4	1
Unusual sensory interest in play material/person	D-1	0
Hand and finger and other complex mannerisms	D-2	0
Excessive interest in or references to unusual or highly specific topics or objects or repetitive behaviors	D-4	1
***RRB total:***	***2***	
**Overall total:**	**9**	

**Table A3:** Results of intellectual functioning using Leiter-3.

**Leiter-3 Subtest**		**Scaled Score**		**Descriptive Classification**
Figure Ground		9		Average
Form Completion		12		Average
Classification and Analogies		14		Above Average
**Leiter-3 Batteries**	**Scaled Score**	**95% CI**	**Percentile Rank**	**Descriptive Classification**
Nonverbal IQ	115	[109, 121]	84	Above Average
Nonverbal Memory	107	[97, 117]	68	Average
Processing Speed	92	[85, 99]	30	Average

**Table A4:** Results of intellectual functioning using UNIT-2.

**UNIT-2 Subtest**		**Scaled Score**		**Descriptive Classification**
Symbolic Memory		9		Average
Nonsymbolic Quantity		12		Average
Analogic Reasoning		11		Average
Spatial Memory		14		Above Average
Numeral Series		10		Average
Cube Design		15		Superior
**UNIT-2 Composite**	**Index Scaled Score**	**95% CI**	**Percentile Rank**	**Descriptive Classification**
Memory	109	[101, 116]	73	Average
Reasoning	117	[111, 122]	87	Above Average
Quantitative	106	[101, 111]	66	Average
***Full Scale Battery***	***113***	***[109, 117]***	***81***	***Above Average***

**Table A5:** Results of adaptive functioning using Vineland-II.

**Vineland-II Domain**	**Standard Score**	**Percentile Rank**	**Descriptive Classification**
Communication	87	19	Adequate
Daily living skills	58	<1	Low, mild deficit
Socialization	58	<1	Low, mild deficit
***Adaptive behavior composite***	***65***	***1***	***Low, mild deficit***
**Vineland-II Maladaptive Domain**	**V-Score**	**Descriptive Classification**	
Internalizing	22	Clinically significant	
Externalizing	19	Elevated	
***Maladaptive behavior index***	***21***	***Clinically significant***	

**Table A6:** Results of adaptive functioning using CBCL/6-18.

Subscale	T-Score	Percentile	Range
**Syndrome Scale**			
Anxious/Depressed	60	84	Normal
Withdrawn/Depressed	60	84	Normal
Somatic Complaints	61	86	Normal
Social Problems	77	>98	Clinical
Thought Problems	66	94	Subclinical
Attention Problems	64	92	Normal
Rule-Breaking Behavior	57	75	Normal
Aggressive Behavior	58	78	Normal
***Internalizing Behavior***	***62***	***-***	***Subclinical***
***Externalizing Behavior***	***58***	***-***	***Normal***
**Total**	**64**	**-**	**Clinical**
**Competence Scale**			
Activities	45	31	Normal
Social	50	50	Normal
School	34	6	Subclinical
**Total**	**43**	**-**	**Normal**
**DSM-Oriented Scale**			
Affective Problems	61	86	Normal
Anxiety Problems	67	94	Subclinical
Somatic Problems	61	84	Normal
Attention Deficit/Hyperactivity Problems	67	95	Subclinical
Oppositional Defiant Problems	58	78	Normal
Conduct Problems	56	72	Normal

**Table A7:** Results of executive function impairment using BRIEF2 (parent’s form, father).

Index/Scale	T-Score	90% CI	Percentile	Descriptive Classification
Inhibit	64	[58; 70]	92	Mildly elevated
Self-Monitor	63	[56; 70]	93	Mildly elevated
*Behavior Regulation Index*	*65*	*[60; 70]*	*91*	*Mildly elevated*
Shift	72	[64; 78]	96	Potentially clinically elevated
Emotional Control	72	[67; 75]	96	Clinically elevated
*Emotion Regulation Index*	*73*	*[69; 77]*	*96*	*Clinically elevated*
Initiate	59	[53; 65]	85	Not elevated
Working Memory	65	[60; 70]	92	Potentially clinically elevated
Plan/Organize	67	[62; 72]	90	Potentially clinically elevated
Task-Monitor	62	[57; 67]	90	Mildly elevated
Organization of Materials	60	[55; 65]	86	Mildly elevated
*Cognitive Regulation Index*	*65*	*[62; 68]*	*90*	*Potentially clinically elevated*
***Global Executive Composite***	***69***	***[67; 71]***	***95***	***Potentially clinically elevated***

**Table A8:** Results of executive function impairment using BRIEF2 (parent’s form, mother).

Index/Scale	T-Score	90% CI	Percentile	Descriptive Classification
Inhibit	67	[61; 73]	94	Potentially clinically elevated
Self-Monitor	74	[81; 67]	99	Clinically elevated
*Behavior Regulation Index*	*70*	*[65; 75]*	*96*	*Clinically elevated*
Shift	76	[70; 82]	98	Clinically elevated
Emotional Control	84	[79; 89]	>99	Clinically elevated
*Emotion Regulation Index*	*83*	*[79; 87]*	*>99*	*Clinically elevated*
Initiate	55	[49; 61]	71	Not elevated
Working Memory	67	[62; 72]	94	Potentially clinically elevated
Plan/Organize	69	[64; 74]	92	Potentially clinically elevated
Task-Monitor	73	[68; 78]	>99	Clinically elevated
Organization of Materials	52	[47; 57]	65	Not elevated
*Cognitive Regulation Index*	*65*	*[62; 68]*	*90*	*Potentially clinically elevated*
***Global Executive Composite***	***76***	***[74; 78]***	***99***	***Clinically elevated***

**Table A9:** Results of executive function impairment using BRIEF2 (self-report form).

Index/Scale	T-Score	90% CI	Percentile	Descriptive Classification
Inhibit	62	[56; 68]	85	Mildly elevated
Self-Monitor	56	[49; 63]	79	Not elevated
*Behavior Regulation Index*	*61*	*[56; 66]*	*83*	*Mildly elevated*
Shift	86	[92; 80]	>99	Clinically elevated
Emotional Control	78	[71; 85]	99	Clinically elevated
*Emotion Regulation Index*	*85*	*[80; 90]*	*>99*	*Clinically elevated*
Task Compilation	61	[55; 67]	84	Mildly elevated
Working Memory	69	[63; 75]	96	Potentially clinically elevated
Plan/Organize	62	[57; 67]	88	Mildly elevated
*Cognitive Regulation Index*	*65*	*[62; 68]*	*90*	*Potentially clinically elevated*
***Global Executive Composite***	***70***	***[67; 73]***	***96***	***Clinically elevated***

**Table A10:** Results of assessment of core symptoms of ADHD using Conners 3.

**Conners 3 Content Scale**		**T-Score**	**Range**
Inattention		59	Average Score
Hyperactivity/Impulsivity		58	Average Score
Learning Problems		71	Very Elevated Score
Executive Functioning		47	Average Score
Aggression		48	Average Score
Peer Relations		73	Very Elevated Score
*Conners-3 Global Index Total*		*59*	*Average Score*
**DSM-5 Symptom Scale**	**T-Score**	**Range**	**Symptom Count** **Requirements**
ADHD Inattention	56	Average Score	1 from 6
ADHD Hyperactivity/Impulsivity	43	Average Score	2 from 6
Conduct Disorder	57	Average Score	0 from 3
Oppositional Defiant Disorder	44	Average Score	1 from 4

## Appendix B

The online databases PubMed and Web of Science were searched for research papers reporting cases of AAS. The following query was applied to titles and abstracts: ‘Faciogenital Dysplasia,’ ‘Faciodigitogenital syndrome,’ ‘Aarskog-Scott Syndrome,’ or ‘Aarskog syndrome.’ This search algorithm was developed based on a previous systematic review [4]. The search was not limited by the year of publication. The initial search of the literature identified a total of 210 records.

The exclusion criteria were as follows: (1) non-full text articles (such as letters and conference theses); (2) studies without original data (i.e., different types of reviews and meta-analyses); (3) studies with averaged data (without personalized information about distinct cases); (4) studies without any information about cognitive and mental development; (5) animal studies; and (6) studies with participants whose age was undefined or lower than 18 months (the minimal age for a reliable diagnosis of ASD [89]). After removing duplicates, two reviewers (RR, OT) screened titles and abstracts against the inclusion criteria. Then, three reviewers (RR, OT, VM) assessed full-text articles for their eligibility for data extraction. In controversial cases, a final decision was made after discussion. Four reviewers (RR, OT, VM, KL) also participated in data extraction. Discrepancies were resolved by consensus or by the first author. During the full-text screening, 141 studies did not meet the eligibility criteria and were excluded (see the diagram above). Ultimately, 64 studies published between 1972 and 2024 were considered (Table S1).
